# Efficacy and Reliability of Mobile Uroflowmetry in Patients With Benign Prostatic Hyperplasia Undergoing Transurethral Resection: Prospective Multicenter Observational Pilot Validation Study

**DOI:** 10.2196/75313

**Published:** 2025-12-05

**Authors:** Sang Hun Song, Younsoo Chung, Hoyoung Ryu, Jeong Woo Lee, Sangchul Lee

**Affiliations:** 1 Department of Urology Seoul National University Bundang Hospital Seongnam-si Republic of Korea; 2 Department of Urology College of Medicine Seoul National University Seoul Republic of Korea; 3 Ewha Womans University Medical Center Seoul Republic of Korea; 4 Department of Urology Kyung Hee University College of Medicine Kyung Hee University Hospital Seoul Republic of Korea

**Keywords:** acoustic uroflowmetry, transurethral resection of prostate, benign prostatic hyperplasia, telemedicine, postsurgical surveillance

## Abstract

**Background:**

Accurate assessment of voiding patterns before and after surgery for lower urinary tract symptoms is critical in patient care, but it places heavy burdens on both the patient and the clinic. While methods for telemedicine have been devised, no technology for acoustic assessment of urinary patterns has been prospectively evaluated for clinical use.

**Objective:**

This study aims to assess the precision of a mobile app–based uroflowmetry and compare it with in-office uroflowmetry measurements for the management of patients undergoing surgical treatment for benign prostatic hyperplasia (BPH).

**Methods:**

This study was designed as a prospective, multicenter, observational pilot validation study conducted at 3 tertiary centers. A total of 46 patients with BPH who had not received any previous treatment within 4 weeks of their initial outpatient clinic visit were prospectively enrolled. After diagnosis, participants with BPH conducted subsequent uroflowmetry measurements by using a sound-based mobile app, proudP, for at least 4 days during the pretreatment period, followed by transurethral resection of prostate (TURP). Additional measurements were taken at the preoperative visit and 4-day periods after 2, 6, and 12 weeks of treatment initiation, with concurrent in-office measurements. Uroflowmetry parameters, including maximum flow rate (Q_max_) and voided volume, were compared. Patient satisfaction was evaluated using a scale ranging from 0 to 10 at the end of the 12-week study.

**Results:**

TURP resulted in a mean Q_max_ improvement of 7.2 mL/s at conventional uroflowmetry, which correlated with a mean improvement of 5.1 mL/s when measured by the app. A statistically significant correlation (*P*<.05) was observed between the 2 methods. The app-based uroflowmetry effectively reflected the improvement in voiding symptoms over time after the initiation of medical treatment, with statistically significant improvement in total International Prostate Symptom Scores (IPSS; –4.7), IPSS obstructive (–5.7), IPSS irritative (–2.6), and quality of life (–5.9; all *P*<.05). Overall, the participants reported a high level of satisfaction, with a mean score of 9.5 (SD 0.8) points at the conclusion of the study.

**Conclusions:**

The findings of this study demonstrate that app-based uroflowmetry (proudP) measurements serve as an accurate and reliable indicator of perioperative surveillance in patients undergoing TURP for BPH. By enabling personalized and portable uroflowmetry, clinicians can easily monitor treatment response as well as observe the risk of postoperative acute urinary retention.

## Introduction

Chronic urinary conditions, such as benign prostatic hyperplasia (BPH), necessitate ongoing patient self-management, akin to other chronic diseases such as hypertension, diabetes, and asthma. Despite this need, there is a notable lack of tools enabling patients to monitor and manage urinary symptoms autonomously at home. This absence increases the risk of symptom progression and the onset of secondary urinary disorders. Even in cases where pharmacological treatments are suboptimal, patients lack effective methods for self-monitoring [1].

Postoperative urinary complications can also occur following interventions for prostate enlargement. The inability of patients to self-assess their condition may result in delayed recognition of issues, potentially necessitating urgent interventions, such as urethral catheterization [2]. Traditional methods, such as the use of a urination diary, which requires patients to record their symptoms for several days using paper and a measuring cup, are cumbersome and often impractical. If these records are misplaced, patients lose the ability to accurately track their urinary habits, and health care professionals face additional burdens in manually calculating metrics such as daily and nocturnal urine output.

The integration of digital therapeutics presents significant benefits, including the absence of toxicity and adverse side effects, minimal costs, and continuous real-time management [3]. These tools facilitate 24-hour monitoring of patient status and allow for personalized patient analytics by empowering patients to actively participate in data collection and management. Individuals can actively engage in self-health monitoring and retrieve evaluation results before consulting medical personnel, reducing time consumed during in-person visits to clinics and overcoming geographic barriers when physical presence is limited due to infection or regional availability of medical services.

As an attempt to address the needs of both clinicians and patients for a personalized device to measure and monitor voiding-related outcomes during and after treatment, an acoustic uroflowmetry was incorporated into a mobile app [4,5]. Uroflowmetry, which is a pivotal test for the evaluation of lower urinary tract function and voiding patterns during diagnosis and treatment, requires the patient to visit the hospital and undergo testing in a controlled setting. Moreover, single measurements obtained in a hospital setting may not reflect a patient’s usual voiding patterns. Acoustic uroflowmetry using mobile apps provides a practical alternative to replace conventional methods, allowing repeated measurements in familiar home environments, enabling remote monitoring as well as accessibility for patients, and improving efficiency in clinical practice.

However, despite worldwide interest and the development of similar tools, no study has generated data on the comparative outcome between mobile uroflowmetry and in-office measurements for surveillance of posttreatment change. This study compares an acoustic analysis–based uroflowmetry, which calculates urine volume by recording sounds with a smartphone, with a conventional physical urinary flow test machine in patients undergoing surgery for BPH. The aim is to evaluate whether traditional in-office tests can be effectively replaced.

## Methods

### Ethical Considerations

This study received approval from the institutional review board of Seoul National University Bundang Hospital (B-2207-769-305). All data analyzed in this research were anonymized, and informed consent was obtained from all patients participating. The participants received monetary compensation of South Korean ₩ 100,000 (approximately US $67.90) for hospital visitation and traveling fees during the entire study. To ensure privacy, personal identifiers were fully removed, and the analysis was conducted anonymously. All procedures adhered to the relevant ethical guidelines and regulations. A STROBE (Strengthening the Reporting of Observational Studies in Epidemiology) checklist was submitted as supplementary material (Multimedia Appendix 1).

### Study Design and Population

Patients diagnosed with BPH planning to undergo surgery (transurethral resection of prostate [TURP]) at 3 tertiary institutions (Seoul National University Bundang Hospital, Ewha Womans University Medical Center, and Kyung Hee University Medical Center), who were older than 20 years, were screened for eligibility and enrolled after obtaining informed consent. This study was designed as a prospective pilot observational study in a single cohort without a control to validate the efficacy of mobile uroflowmetry measurements compared to conventional methods conducted in an office. On the basis of previous literature [4,5] suggesting a moderate correlation (expected *r*=0.6), a minimum of 20 patients would provide 80% power to detect a statistically significant correlation at a 2-sided α level of .05. To improve the precision of the correlation estimate, allow for potential measurement failure or incomplete data, and support the feasibility of future definitive studies, we increased the target enrollment to 40 patients. During the first outpatient visits, patients planning to undergo surgery for BPH were recommended for screening and enrollment, if eligible. After initial study enrollment, individual mobile devices with the app installed were distributed for preoperative evaluation of voiding patterns and parameter measurements for at least 72 to 96 hours before surgery. In-office measurements were also conducted for comparison. Additional app measurements were taken for the same 4-day periods after 2, 6, and 12 weeks of surgery and compared to conventional uroflowmetry measurements conducted at the same intervals of outpatient clinic visits. International Prostate Symptom Scores (IPSS) and uroflowmetry parameters, including maximum flow rate (Q_max_) and voided volume (VV), were collected at each visit. At the time of study termination at 12 weeks, all patients completed a written survey using a 0-to-10-point scale for satisfaction. All enrolled participants completed the study protocol, and there were no missing data for any of the outcome variables over the 12-week follow-up period.

### In-Office and Mobile App–Based Uroflowmetry

All patients uniformly underwent an in-office conventional uroflowmetry (CubeFlow_S; MCube Technology) at each visit before and after treatment according to the study schedule. Additional app-based uroflowmetry measurements were obtained using the sound-based mobile app proudP (Soundable Health, Inc), a Food and Drug Administration–listed class 2 uroflow meter that has been validated for flow prediction and VV measurements in previous studies [4,6]. The acoustic flow measurement system uses a wireless, smartphone-based approach with recording capabilities to analyze urinary flow. Acoustic data were captured in real time using a smartphone app. From the recorded sounds, parameters such as urinary volume, flow-related metrics (eg, peak urinary flow and average urinary flow), urinary flow patterns (eg, continuous or intermittent), and time-related parameters (eg, maximum voiding time and voiding duration) were calculated. Acoustic characteristics were assessed using audio processing, signal preprocessing, and spectral analysis techniques. A predictive model was then used to estimate urinary flow and associated parameters. Postprocessing produced data regarding accuracy and voiding metrics.

To minimize variability due to height or ambient surrounding noise, patients were instructed to ensure the restroom environment was as quiet as possible and place their smartphone approximately 80 cm away from the toilet before using the mobile app. They were then guided to launch the mobile uroflowmetry. While standing in front of the toilet, patients urinated, aiming for the center of the bowl, when possible, to optimize measurement accuracy. Before the actual experiment, all participants received standardized instructions on how to perform uroflowmetry using the in-hospital system to ensure consistent and reliable data collection.

The recorded sounds were analyzed using audio editing software (Audacity, version 2.2.2; Audacity Open Source Team and GoldWave). Signal preprocessing and postprocessing as well as the development of flow prediction models were conducted using MATLAB (version R2017b, 9.3.0; MathWorks, Inc) and Python (Python Software Foundation). To enhance accuracy, the acoustic analysis algorithm included preprocessing and postprocessing refinements to eliminate short-term artifacts and outliers, correct background noise levels, and remove specific noise bands. Validation of uroflowmetry parameters, including Q_max_ and VV, was performed in separate studies [6].

### Survey Method

To assess patient satisfaction with the mobile uroflowmetry system, we developed a brief, study-specific questionnaire tailored for use in this pilot validation study, and a single-session self-administered survey was conducted at the final in-office visit. The questionnaire was created collaboratively by the study investigators, including urologists and research coordinators, based on their clinical experience and anticipated domains of usability (eg, convenience, clarity of instructions, and perceived reliability of the mobile app). Patients were asked whether the use of the app-based monitoring (1) allowed better self-understanding of their clinical status, (2) improved the clinician’s understanding of their status, (3) was easy to use, and (4) was overall satisfactory. All scores were provided as a numerical value on a point scale ranging from 0 to 10.

### Statistical Analysis

Independent 2-tailed *t* tests and equal-variance tests were used to assess whether there were statistically significant differences between conventional measurements obtained via in-office uroflowmetry–based and acoustic uroflowmetry–based mobile data collection. These tests were selected to validate the statistical characteristics of uroflowmetry measurements, including Q_max_ as the primary comparative factor, with VV and IPSS change as secondary measures. The analysis and calculations were conducted using Python (version 3.6.9; Python Software Foundation), along with the SciPy (version 1.5.14; Python Software Foundation) scientific computing package and R (version 4.3.1; R Foundation for Statistical Computing). Categorical variables were analyzed with chi-square and Fisher exact tests, and ANOVA was used for additional continuous variables. Normality was assessed using the Shapiro-Wilk test, and homogeneity of variance was evaluated with the Levene test. In cases where assumptions were not met, appropriate nonparametric alternatives (eg, Mann-Whitney *U* test and Kruskal-Wallis test) were used. Further assessment of the agreement of the 2 different uroflowmetry parameters was performed with Bland-Altman analysis, with the mean difference and 95% limits of agreement (defined as mean difference of +1.96 and –1.96 × SD of the paired differences) calculated according to standard procedures. To interpret clinical relevance, we adopted a provisional threshold of +10 mL/s and –10 mL/s for Q_max_, as variations of this magnitude are generally regarded as unlikely to change the clinical interpretation of flow pattern or degree of obstruction in typical urodynamic practice [7].

## Results

**Figure 1:**
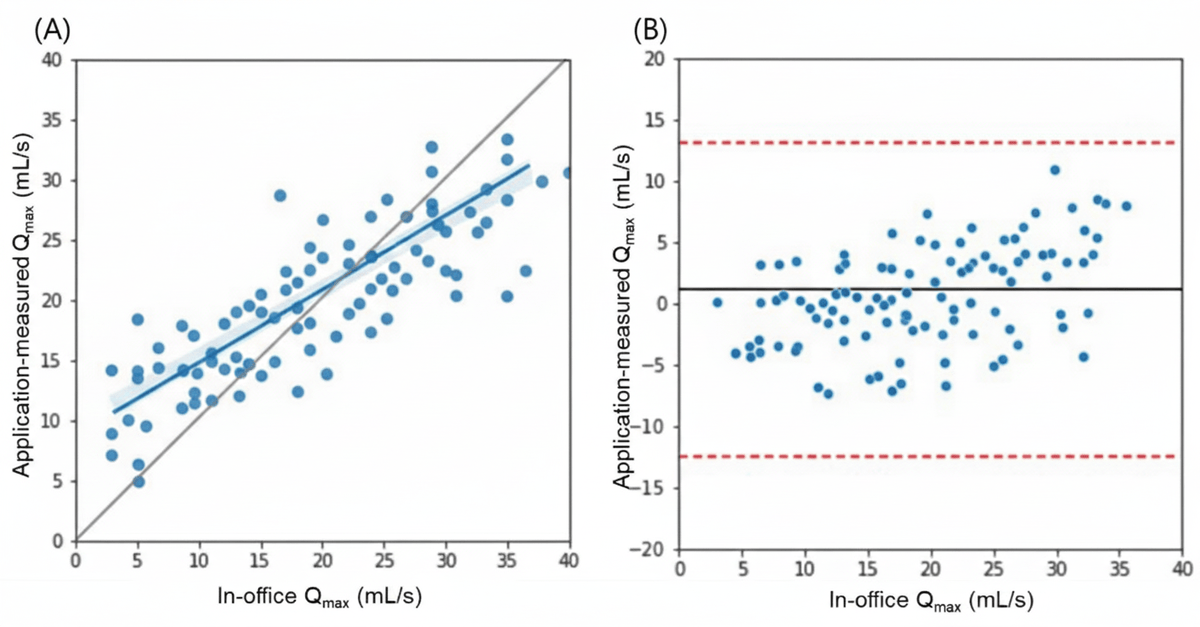
(A) Correlation and (B) Bland-Altman analysis of in-office and app-measured maximum flow rate (Qmax). Regression lines with 95% CIs are depicted in light blue, and mean bias (gray) and 95% upper and lower limits of agreement (dashed red) are displayed in horizontal lines.

A total of 46 treatment-naive patients with symptomatic BPH undergoing endoscopic surgery were screened, and 41 (89%) patients were finally enrolled, with 5 (11%) declining participation. The mean age of all patients was 67.4 (SD 5.5; range 58-79) years. Assessment of the accuracy and representability of acoustic uroflowmetry as compared to in-office measurements for Q_max_ resulted in a Pearson correlation of 0.743 (*P*<.001; Figure 1). The mean of the difference observed in the Bland-Altman analysis was 1.57 (SD 7.0), with upper and lower limits of agreement of 15.4 and –12.2, respectively.

Improvement in IPSS over the 12-week period was significant for all patients who underwent TURP for all specific parameters, including total IPSS and quality of life, as well as for both obstructive and irritative symptoms (Table 1; Figure S1 in Multimedia Appendix 2). Mean improvement of 10.2 and 8 points was observed for total IPSS and obstructive IPSS, respectively (both *P*<.001).

**Table 1:** Perioperative International Prostate Symptom Score (IPSS) change.

	Baseline, mean (SD)	2 weeks, mean (SD)	6 weeks, mean (SD)	12 weeks, mean (SD)	*P* value
IPSS total	18.0 (8.0)	12.1 (8.1)	10.3 (7.1)	7.8 (6.6)	<.001
IPSS obstructive	10.7 (5.1)	5.2 (5.4)	3.5 (4.1)	2.7 (4.0)	<.001
IPSS irritative	7.3 (3.1)	6.9 (3.6)	6.8 (3.7)	5.2 (3.4)	.009
IPSS quality of life	4.2 (0.8)	2.3 (1.9)	2.3 (1.7)	1.8 (1.5)	<.001

Specific uroflowmetry parameters, as measured from the mobile device, well reflected improvements in symptom scores, with baseline mean Q_max_ of 12.8 (SD 4.1) improving to 20.3 (SD 5.4) at the end of the study, similar to results measured from in-office uroflowmetry, which showed a similar range of improvement from a mean of 13.0 (SD 6.3) to 23.2 (SD 9.8) in the same period (Table 2). No significant differences in VV were observed.

**Table 2:** Perioperative change in maximum flow rate (Qmax) and voided volume as measured by conventional in-office uroflowmetry and app-based uroflowmetry.

Parameter	In-office uroflowmetry					App-based uroflowmetry			
	Baseline	2 weeks	6 weeks	12 weeks	Baseline		2 weeks	6 weeks	12 weeks
Q_max_ (mL/s), mean (SD)	13.7 (6.0)	21.4 (10.6)	22.0 (10.3)	20.9 (10.5)	14.1 (5.0)		18.3 (4.9)	20.0 (6.0)	19.2 (6.4)
Voided volume (mL), mean (SD)	221 (109)	215 (135)	203 (145)	189 (102)	261 (94)		242 (79)	215 (79)	237 (90)
Posttransurethral resection of prostate change in Q_max_ (mL/s)	Reference	7.7	8.5	7.2	Reference		4.2	5.9	5.1

**Figure 2:**
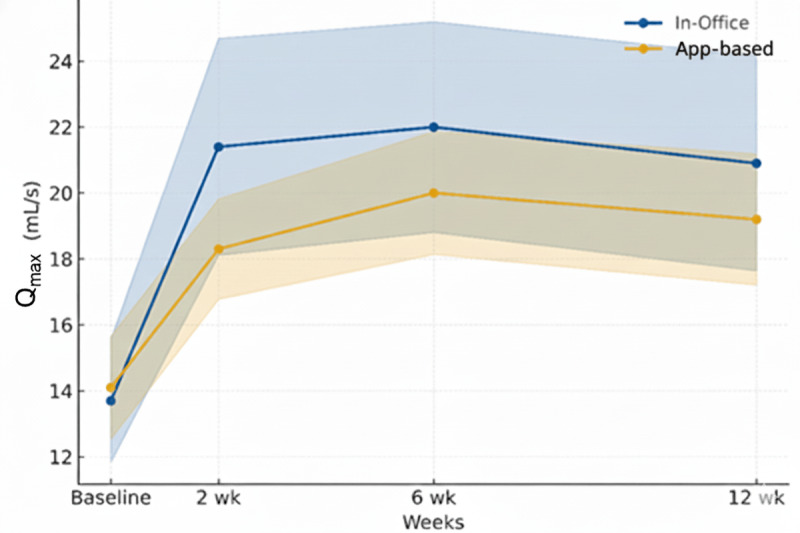
Longitudinal maximum flow rate (Qmax) change before and after treatment initiation.

Changes in IPSS to Q_max_ as measured by the app were significant overall, with modest correlation (Figure 2) and the highest Pearson correlation of –0.490 for obstructive IPSS, followed by –0.478 for total IPSS (Figure S2 in Multimedia Appendix 2). Individual questions for intermittency and weak stream showed the highest correlation as measured by acoustic uroflowmetry (*r*=–0.490 and *r*=–0.580, respectively).

When stratified by prostate volume, patients with larger prostates (≥80 mL) demonstrated greater improvement in Q_max_ at 12 weeks compared to those with smaller prostates (<80 mL). Mean Q_max_ in the 80 mL or greater prostate volume group increased from 13.1 (SD 4.8) mL/s at baseline to 22.8 (SD 4.4) mL/s at 12 weeks using the app-based method compared with a 10.1 mL/s increase measured by in-office uroflowmetry. In contrast, patients with prostate volumes less than 80 mL showed a smaller mean increase (app: mean 13.0, SD 4.0 to mean 19.4, SD 5.3 mL/s; in office: mean 12.6, SD 5.3 to mean 22.8, SD 11.9 mL/s). Correlation between the 2 methods was also slightly higher in the larger prostate subgroup (*r*=0.692) than in the smaller prostate group (*r*=0.642; both *P*<.001), suggesting more consistent agreement in men with enlarged glands (Table S1 in Multimedia Appendix 2).

Further stratification by severity of IPSS showed both improvements reflected in patients with either moderate or severe IPSS, with a high Pearson correlation value of 0.751 and 0.734 in each group (all *P*<.001; Figure S3 in Multimedia Appendix 2).

Survey results for patient satisfaction are shown in Table 3. All patients were highly satisfied with the measurement process and felt the app was easy to use. Subjective assessment of the additive value of the app was remarkably high. No difference in the use of the app by men aged 70 years and older was observed.

**Table 3:** Patient satisfaction survey.

Survey item	Scores of all patients, mean (SD)	Scores of those aged ≥70 years, mean (SD)
Can better assess my own clinical status	9.4 (1.2)	9.1 (1.2)
Can improve my physician’s assessment of my status	9.4 (1.7)	9.7 (0.5)
Was convenient and easy to use	9.4 (1.1)	9.7 (0.7)
Overall satisfaction	9.4 (0.9)	9.3 (0.7)

**Figure 3:**
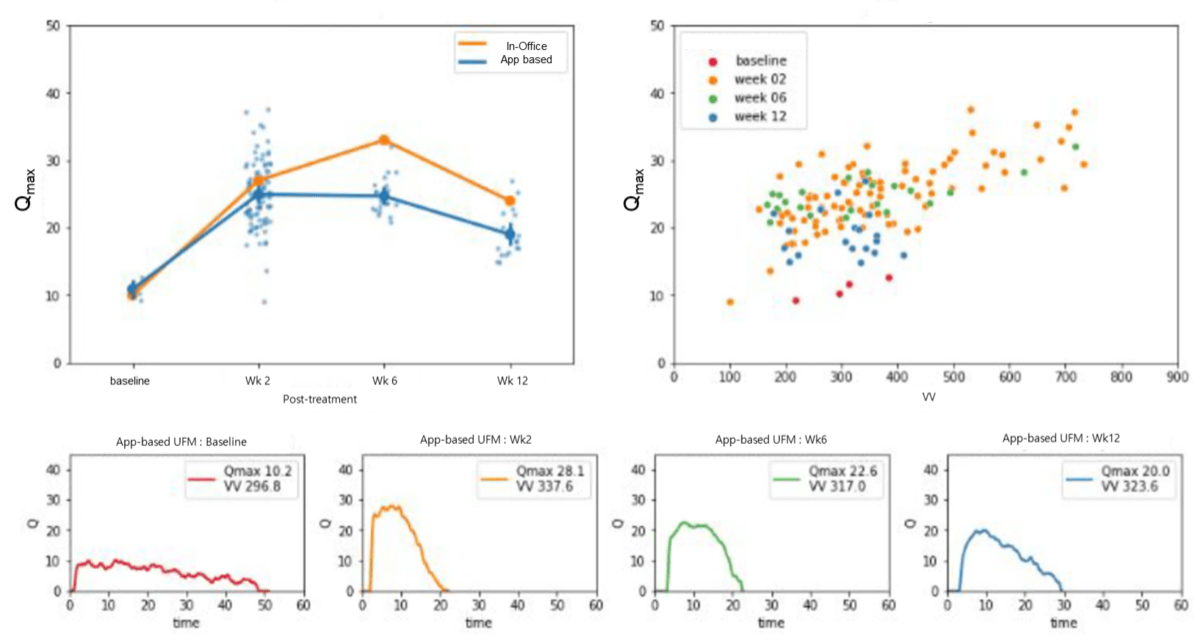
Posttransurethral resection of prostate representation in a single patient. Qmax: maximum flow rate; UFM: uroflowmetry; VV: voided volume.

A sample representation of post-TURP changes and measurements conducted with the mobile uroflowmetry is presented in Figure 3, where a patient’s preoperative obstructive patterns and postoperative improvement of uroflowmetry plateau are well displayed, with an initial Q_max_ of 10.2 and VV of 297 improved to 20.0 and 324, respectively, at 12 weeks after surgery.

## Discussion

### Principal Findings

This is the first prospective clinical trial to evaluate the effectiveness and feasibility of an acoustic app-based uroflowmetry to monitor patients after clinical intervention. The mobile measurements conducted at home were clinically reliable, with a strong correlation to IPSS improvement after surgery, also reliably reflecting the absolute improvement in Q_max_ with TURP, especially for patients with obstructive IPSS. Q_max_, as measured with the app, showed consistent change regardless of prostate size as well as when stratified by severity of IPSS, suggesting that the technology can be reliably used in a wide spectrum of male patients with lower urinary tract symptoms. Older patients were equally satisfied with the process and felt at ease using the app, suggesting that as long as the patient is familiar with a mobile device, uroflowmetry measurements for clinical observation can be effectively conducted without technical difficulty.

Lee et al [8] performed a prospective comparative analysis in 16 male pediatric patients using the same app to validate the technology’s strong correlation to standard measurements. A smartwatch-based uroflowmetry model was constructed by a Spanish team after extracting acoustic features from voiding stream sounds, similar to the method described in this study, and it displayed good correlation [9]. El Helou et al [10] used a similar approach in 44 healthy young men, and by mapping total sound energy with VV, the model was successfully able to estimate flow rate with a mean absolute error of 2.41 mL/s. Dawidek et al [11] compared a conventional uroflowmetry with an audio-based uroflowmetry (TeleSonoUroFlow) app, achieving a poor correlation for Q_max_ (*r*=0.12) and failing to show consistent results despite modest improvement in healthy individuals. However, these studies, by design, performed only comparisons of the mobile uroflowmetry versus conventional clinic-based measurements and did not evaluate whether the technology could accurately represent the changes that occur during and after treatment. Overall, a recent meta-analysis by Rangganata et al [12] showed the efficacy of mobile acoustic uroflowmetry in male participants to be strong, with positive correlation for VV and Q_max_, as shown in our study, as well as for other uroflowmetry parameters, including voiding time and average flow. Bladt et al [13] also showed that at-home measurements can be as useful or even more representative of voiding patterns than hospital measurements, as shown in our sample patient (Figure 3), in whom multiple app-based measurements were more informative and reliable in tracking voiding pattern changes.

The importance of uroflowmetry and changes in its parameters are paramount in assessing the success and efficacy of surgical treatment in BPH [14-16]. While preoperative Q_max_ values typically range from 6.18 to 8 mL/s, Q_max_ improves significantly after surgery, with studies reporting improvement up to 26.43 mL/s [17,18]. The average flow rate shows similar changes, with preoperative values from approximately 4.44 to 13.48 mL/s after TURP [19]. The patients in our cohort showed similar improvement, with most change found in large BPH. The significance of our study lies in the fact that mobile uroflowmetry was sufficient to measure the changes in such parameters after surgical intervention and reflect the measurements performed at outpatient visits, validating the efficacy for use in actual clinical practice. Unlike previous studies that primarily validated acoustic uroflowmetry in healthy volunteers or patients with stable lower urinary tract symptoms, this study evaluated its performance in a postoperative population undergoing active recovery after prostate surgery. In this context, uroflow parameters fluctuate considerably due to progressive relief of obstruction, healing of the bladder neck, and adaptation of detrusor contractility. Demonstrating consistent agreement between acoustic and conventional measurements across this dynamic postoperative trajectory supports the robustness of acoustic uroflowmetry beyond static or screening scenarios. Therefore, our findings extend the clinical applicability of this technology to longitudinal monitoring in the perioperative setting, where repeated, home-based assessments can provide meaningful insights into functional recovery. However, while tracking changes was significantly well correlated, our results suggest caution when considering complete replacement of conventional measurements, as the limits of agreement in this study (–12.2 to 15.4 mL/s) slightly exceed the prespecified reference range of +10 mL/s and –10 mL/s, suggesting that although the 2 devices show close overall agreement, individual measurements may differ modestly in real-world clinical use. This difference may reflect consistent measurement conditions at home despite pretraining and guidance during the trial or may result from intraindividual variability in uroflowmetry, which by itself is known to reach approximately 10 mL/s [20]. This finding highlights the need and necessity for multiple measurements in a single individual during clinical evaluation and monitoring, underscoring the importance and potential for remote mobile measurements.

Another interesting point to mention was that a stronger agreement was observed in men with larger prostates or higher baseline IPSS. This may reflect the more stable and reproducible flow characteristics typically seen in obstructive voiding patterns, in which urinary flow is typically slower and of longer duration, producing clearer and less noisy acoustic signals that enhance the reliability of the app’s waveform detection. Conversely, individuals with smaller prostates or milder symptoms often exhibit higher peak variability and shorter flow times, which can amplify measurement discrepancies between acoustic and conventional methods. These subgroup findings suggest that app-based uroflowmetry may be particularly accurate for monitoring patients with clinically significant bladder outlet obstruction, while careful interpretation is still warranted in those with near-normal flow profiles.

Beyond demonstrating technical validity, this study also highlights the digital health potential of acoustic uroflowmetry. The app-based measurement system allows patients to record voiding data conveniently without additional equipment, such as measuring cups or paper logs. Previous studies have reported higher satisfaction and adherence with app-based systems compared to conventional uroflowmetry, even among older adults unfamiliar with smartphones [16]. In our cohort, similar usability was observed among participants aged more than 70 years, likely reflecting both the intuitive interface design and the brief in-office education that enhanced confidence and accuracy of use [21,22]. The automatic generation of an electronic voiding diary may have further increased engagement by reducing manual documentation and simplifying self-tracking.

From a clinical workflow perspective, such usability supports integration of acoustic uroflowmetry into telemedicine and self-management pathways for BPH and postoperative monitoring. Home-based acoustic measurements can be securely transmitted to clinicians for asynchronous review, enabling continuous monitoring of recovery trends and early identification of voiding deterioration without frequent in-person visits. When combined with patient-reported outcomes, such as IPSS, app-based flow metrics may enhance remote clinical decision-making and support personalized treatment adjustments. Integration with electronic medical records and automated alerts based on individualized thresholds could further streamline care within digital urology ecosystems.

Nevertheless, several equity and accessibility considerations should be acknowledged. Smartphone literacy remains a potential barrier, particularly among older or socioeconomically disadvantaged populations. In our study, targeted education and a simplified user interface mitigated many of these challenges, but broader implementation will require interfaces accommodating sensory or cognitive limitations. Cost and device availability also remain relevant, as not all patients may have access to compatible smartphones or stable internet connections, potentially widening digital health disparities. Data privacy and cybersecurity represent additional priorities. Because acoustic recordings and voiding profiles constitute personal health information, strict adherence to encryption, anonymization, and data protection regulations (eg, General Data Protection Regulation and Health Insurance Portability and Accountability Act) is essential. Finally, regulatory approval processes for software as a medical device must be clearly defined to ensure safety, performance, and clinical accountability. Early engagement with regulatory authorities and compliance with international validation frameworks will be crucial for widespread clinical adoption.

Collectively, these findings suggest that app-based uroflowmetry not only provides accurate and reproducible measurements but also aligns with the evolving paradigm of patient-centered, connected urological care. With careful attention to usability, privacy, and equity, such technologies could substantially enhance accessibility to postoperative monitoring and chronic symptom management through scalable digital health integration.

### Limitations

This study is not without limitations. First, conventional uroflowmetry was performed at a single session, with repeat measurements conducted only if the patient was unable to void at the first trial or had low VV (≤150 mL), to ensure reliability of the uroflowmetry measurements. However, a single in-office voiding trial may overestimate or underestimate the actual symptoms and change over clinical course, and repeated measurements, as in the mobile uroflowmetry, may be required. Second, no information on TURP clinical variables, such as baseline prostate-specific antigen, resection volume, or pathology, was included in our analysis. While the loss of such information was detrimental to our results, resection percentage as a surrogate for completeness of adenoma resection may have shown a strong correlation with IPSS and Q_max_ change as estimated from both app-based and in-office measurements, supporting our findings. Moreover, as this study aimed to validate agreement between mobile and in-office uroflowmetry, any such factors would likely have affected both modalities equally and thus are unlikely to alter the comparative findings. Given the exploratory nature of this study, survey questionnaires were custom made and were not validated in a separate study, which may undermine the reliability of the reported outcomes. Limitations associated with the lack of psychometric validation are acknowledged, and future studies should incorporate standardized and validated patient-reported outcome measures to strengthen the assessment of usability. Finally, this study did not include a randomized control group and was conducted in a pilot prospective observational trial setting, limiting the strength of causal inference. However, this design was deliberately chosen to evaluate the feasibility and assess the preliminary performance of the mobile uroflowmetry in a clinical treatment scenario, with the plan of performing larger studies in a randomized and controlled framework to establish the potential replacement of conventional methods. The limited sample size and observational design of this study suggest potential for mobile uroflowmetry; however, they are insufficient to fully support complete replacement of conventional uroflowmetry. Future head-to-head randomized controlled trials comparing long-term outcomes between mobile uroflowmetry and in-office measurements are required to further validate the clinical efficacy of mobile methods. In particular, appropriate methods, such as Bonferroni or false discovery rate correction to address multiple testing and repeated measures, will be required.

Taken together, this study demonstrates the feasibility of using mobile, app-based uroflowmetry as a reliable alternative to conventional in-office measurements. By overcoming the spatial and temporal limitations inherent to traditional uroflowmetry, app-based measurements enable continuous, home-based assessment of postoperative urinary flow dynamics. Unlike previous validation studies limited to healthy or stable populations, our findings extend the applicability of this technology to a postsurgical cohort, showing its potential to capture dynamic recovery patterns and detect functional improvement without requiring frequent outpatient visits. Although no cases of acute retention or early stricture occurred during follow-up, the ability to remotely monitor flow changes suggests a role for early detection of postoperative complications and personalized recovery tracking.

Nonetheless, these findings should be interpreted within the scope of a feasibility study. This work establishes proof of concept and short-term clinical reliability but does not yet address long-term adherence, scalability, or outcome-driven end points. The logical next steps include conducting a randomized controlled trial comparing acoustic and conventional uroflowmetry in diverse clinical settings, followed by broader real-world implementation studies to evaluate cost-effectiveness, user engagement, and system integration within telehealth and electronic medical record platforms. With such validation, app-based uroflowmetry could evolve into a scalable, patient-centered component of precision urological care.

### Conclusions

In this prospective pilot observational study, app-based uroflowmetry (proudP) measurements showed reasonable concordance with conventional in-office testing, indicating its feasibility as a tool for perioperative surveillance in BPH surgery. The app-based system effectively reflected both in-office flow values and longitudinal changes in symptom severity, as measured by IPSS, suggesting potential for reliable home-based monitoring of postoperative recovery. Nonetheless, the absence of randomization, the use of a single cohort, and the limited follow-up necessitate caution in interpreting these findings. Future large-scale randomized and real-world implementation studies across diverse populations with lower urinary tract symptoms are warranted to establish the clinical validity, cost-effectiveness, and scalability of app-based uroflowmetry as a practical extension of telemedicine in contemporary urological care.

## Data Availability

The datasets generated or analyzed during this study are available from the corresponding author on reasonable request.

## Appendix

Multimedia Appendix 1 STROBE (Strengthening the Reporting of Observational Studies in Epidemiology) checklist.

Multimedia Appendix 2 Analyses of IPSS (International Prostate Symptom Scores) and Qmax (maximum flow rate) changes and correlations.

## Abbreviations

BPH: benign prostatic hyperplasia
IPSS: International Prostate Symptom Scores
Qmax: maximum flow rate
STROBE: Strengthening the Reporting of Observational Studies in Epidemiology
TURP: transurethral resection of prostate
VV: voided volume
