# Supplementary material for: Efficacy and Reliability of Mobile Uroflowmetry in Patients With Benign Prostatic Hyperplasia Undergoing Transurethral Resection: Prospective Multicenter Observational Pilot Validation Study
Source: J Med Internet Res. 2025 Dec 5;27:e75313. doi: 10.2196/75313 (PMC12717504; doi:10.2196/75313)
Supplement: Multimedia Appendix 1 [file jmir_v27i1e75313_app1.pdf]

STROBE Statement—checklist of items that should be included in reports of observational studies

	Item No.	Recommendation	Page No.	Relevant text from manuscript
**Title and abstract**	1	(*a*) Indicate the study's design with a commonly used term in the title or the abstract	1	"…prospective multicenter clinical trial"
		(*b*) Provide in the abstract an informative and balanced summary of what was done and what was found	2	Background-Objectives-Materials and methods-Results-Conclusion
**Introduction**				
Background/rationale	2	Explain the scientific background and rationale for the investigation being reported	4	"However, despite worldwide interest and generation of similar tools, no study has generated data on the comparative outcome between mobile UFM and in-office measurements for surveillance of post-treatment change."
Objectives	3	State specific objectives, including any prespecified hypotheses	5	"The aim is to evaluate whether traditional in-office tests can be effectively replaced."
**Methods**				
Study design	4	Present key elements of study design early in the paper	6	Study design and population section
Setting	5	Describe the setting, locations, and relevant dates, including periods of recruitment, exposure, follow-up, and data collection	6-8	
Participants	6	(*a*) *Cohort study*—Give the eligibility criteria, and the sources and methods of selection of participants. Describe methods of follow-up<br>*Case-control study*—Give the eligibility criteria, and the sources and methods of case ascertainment and control selection. Give the rationale for the choice of cases and controls<br>*Cross-sectional study*—Give the eligibility criteria, and the sources and methods of selection of	6-7	"Patients diagnosed with BPH planning to undergo surgery (transurethral resection of prostate) at three tertiary institutions (Seoul National

		participants		University Bundang Hospital, Ewha Womans University Medical Center, and Kyung Hee University Medical Center) over the age of 20 years were screened for eligibility and enrolled after informed consent…”
		(*b*) *Cohort study*—For matched studies, give matching criteria and number of exposed and unexposed<br>*Case-control study*—For matched studies, give matching criteria and the number of controls per case		
Variables	7	Clearly define all outcomes, exposures, predictors, potential confounders, and effect modifiers. Give diagnostic criteria, if applicable	7	“International prostate symptom scores (IPSS), UFM paramenters including maximum flow rate (Qmax) and voided volume (VV)…” acoustic UFM parameters discussed
Data sources/ measurement	8*	For each variable of interest, give sources of data and details of methods of assessment (measurement). Describe comparability of assessment methods if there is more than one group	*7-9*	*In-office and mobile application-based uroflowmetry, Survey method, and Statistical analysis section*
9Bias	9	Describe any efforts to address potential sources of bias	9	“Normality was assessed using the Shapiro–Wilk test, and homogeneity of variance was evaluated with Levene’s test. In cases where assumptions were not met, appropriate nonparametric alternatives (e.g., Mann–Whitney U test, Kruskal–

				Wallis test) were used.”
Study size	10	Explain how the study size was arrived at	6	“Based on prior literature [4,5] suggesting a moderate correlation (expected r = 0.6), a minimum of 20 patients would provide 80% power to detect a statistically significant correlation at a two-sided alpha level of 0.05. To improve the precision of the correlation estimate, allow for potential measurement failure or incomplete data, and support the feasibility of future definitive studies, we increased the target enrollment to 40 patients”

Continued on next page

Quantitative variables	11	Explain how quantitative variables were handled in the analyses. If applicable, describe which groupings were chosen and why	8-9	"Independent t-tests and equal-variance tests were employed to assess whether there was statistical evidence indicating significant differences between conventional measurements via In-office UFM and acoustic UFM-based mobile data collection. These tests were selected to validate the statistical characteristics of UFM measurements including Qmax as the primary comparative factor, with VV and IPSS change as secondary measures."
Statistical methods	12	(*a*) Describe all statistical methods, including those used to control for confounding	8-9	"Independent t-tests and equal-variance tests…. In cases where assumptions were not met, appropriate nonparametric alternatives (e.g., Mann–Whitney U test, Kruskal–Wallis test) were used."
		(*b*) Describe any methods used to examine subgroups and interactions	8-9	"Categorical variables were analyzed with chi-squared and Fisher's exact test, and ANOVA was used for additional continuous variables."
		(*c*) Explain how missing data were addressed	7	"All enrolled participants after screening completed the study protocol, and there were no missing data for any of the outcome variables over the 12-week follow-

				up period.”
		(*d*) *Cohort study*—If applicable, explain how loss to follow-up was addressed<br>*Case-control study*—If applicable, explain how matching of cases and controls was addressed<br>*Cross-sectional study*—If applicable, describe analytical methods taking account of sampling strategy	7	“All enrolled participants after screening completed the study protocol, and there were no missing data for any of the outcome variables over the 12-week follow-up period.”
		(*e*) Describe any sensitivity analyses	N/A	
**Results**				
Participants	13*	(a) Report numbers of individuals at each stage of study—eg numbers potentially eligible, examined for eligibility, confirmed eligible, included in the study, completing follow-up, and analysed	10	“Total 46 treatment-naïve patients with symptomatic BPH undergoing endoscopic surgery were screened, and 41 patients were finally enrolled with 5 declining participation”
		(b) Give reasons for non-participation at each stage	10	“Total 46 treatment-naïve patients with symptomatic BPH undergoing endoscopic surgery were screened, and 41 patients were finally enrolled with 5 declining participation”
		(c) Consider use of a flow diagram	N/A	
Descriptive data	14*	(a) Give characteristics of study participants (eg demographic, clinical, social) and information on exposures and potential confounders	9	“Mean age of all patients was 67.4±5.5 years old (range 58-79).”
		(b) Indicate number of participants with missing data for each variable of interest	7	“All enrolled participants after screening completed the study protocol, and there were no missing data for any of the outcome variables over the 12-week follow-up period.”
		(c) *Cohort study*—Summarise follow-up time (eg, average and total amount)	N/A	12weeks

Outcome data	15*	*Cohort study*—Report numbers of outcome events or summary measures over time	*9-11*	*Results section*
		Case-control study—Report numbers in each exposure category, or summary measures of exposure		
		Cross-sectional study—Report numbers of outcome events or summary measures		
Main results	16	(*a*) Give unadjusted estimates and, if applicable, confounder-adjusted estimates and their precision (eg, 95% confidence interval). Make clear which confounders were adjusted for and why they were included	*9-11*	*Results section*
		(*b*) Report category boundaries when continuous variables were categorized	*10-11*	"When stratified by prostate volume, patients with preoperative PV above 80ccs…", "Further stratification by severity of IPSS showed both improvement reflected in patients with either moderate or severe IPSS…"
		(*c*) If relevant, consider translating estimates of relative risk into absolute risk for a meaningful time period	N/A	

Continued on next page

Other analyses	17	Report other analyses done—eg analyses of subgroups and interactions, and sensitivity analyses	10-11	"When stratified by prostate volume, patients with preoperative PV above 80ccs…", "Further stratification by severity of IPSS showed both improvement reflected in patients with either moderate or severe IPSS…"
**Discussion**				
Key results	18	Summarise key results with reference to study objectives	11	"This is the first prospective clinical trial to evaluate the effectiveness and feasibility of an acoustic application-based UFM to monitor patients after clinical intervention. The mobile measurements conducted at home were clinically reliable with strong correlation to IPSS improvement after surgery, also reliably reflecting the absolute improvement in Qmax with TURP especially for patients with obstructive IPSS. Qmax as measured with app showed consistent change regardless of prostate size as well as when stratified by severity of IPSS, suggesting that the technology can be reliably utilized in a wide spectrum of patients with male LUTS. Elderly patients were equally satisfied with the process and felt at ease using the

				application, suggesting that as long as the patient is familiar with a mobile device, UFM measurements can be effectively conducted without risk of technical difficulty.
Limitations	19	Discuss limitations of the study, taking into account sources of potential bias or imprecision. Discuss both direction and magnitude of any potential bias	13	"This study is not without limitations…"
Interpretation	20	Give a cautious overall interpretation of results considering objectives, limitations, multiplicity of analyses, results from similar studies, and other relevant evidence	14	"Nonetheless, this study indicates that mobile UFM can be an effective alternative for in-office measurements that overcome the limitations of space and time delay required for conventional UFM. While previous studies have focused on comparative analysis of UFM measurements taken from healthy populations, our study was successfully able to show the efficacy of mobile UFM in monitoring post-surgical change in UFM parameters without the need for frequent visits to the outpatient clinic. While no case of acute retention or immediate stricture during the postoperative surveillance period were observed in our cohort, the technology has the potential to detect early complications with at-home measurements and provide personalized postoperative monitoring without further

				increasing frequency of visits to the local clinic.”
Generalisability	21	Discuss the generalisability (external validity) of the study results	14	“the technology has the potential to detect early complications with at-home measurements and provide personalized postoperative monitoring without further increasing frequency of visits to the local clinic.”
**Other information**				
Funding	22	Give the source of funding and the role of the funders for the present study and, if applicable, for the original study on which the present article is based	15	“This work was supported by the Korea Medical Device Development Fund grant funded by the Korea government (the Ministry of Science and ICT, the Ministry of Trade, Industry and Energy, the Ministry of Health & Welfare, the Ministry of Food and Drug Safety) (Project Number: 1711138269 , RS-2020-KD000141) (NTIS, RS-2020-KD000141) and by grant no 14-2021-0021 from SNUBH Research Fund.”

*Give information separately for cases and controls in case-control studies and, if applicable, for exposed and unexposed groups in cohort and cross-sectional studies.

Note: An Explanation and Elaboration article discusses each checklist item and gives methodological background and published examples of transparent reporting. The STROBE checklist is best used in conjunction with this article (freely available on the Web sites of PLoS Medicine at http://www.plosmedicine.org/, Annals of Internal Medicine at http://www.annals.org/, and Epidemiology at http://www.epidem.com/). Information on the STROBE Initiative is available at www.strobe-statement.org.
