# Supplementary material for: Efficacy and Reliability of Mobile Uroflowmetry in Patients With Benign Prostatic Hyperplasia Undergoing Transurethral Resection: Prospective Multicenter Observational Pilot Validation Study
Source: J Med Internet Res. 2025 Dec 5;27:e75313. doi: 10.2196/75313 (PMC12717504; doi:10.2196/75313)

Table S1. Qmax change stratified by prostate size

	Application				In-office			
Mean Qmax (mL/sec)	Baseline	2WK	6WK	12WK	Baseline	2WK	6WK	12WK
TRUS<80	13.0 ± 4.0	18.4 ± 4.9	20.0 ± 4.3	19.4 ± 5.3	12.6 ± 5.3	23.0 ± 8.9	20.5 ± 8.9	22.8 ± 11.9
TRUS≥80	13.1 ± 4.8	20.3 ± 4.6	23.8 ± 4.6	22.8 ± 4.4	14.2 ± 6.2	25.2 ± 11.1	28.2 ± 9.4	24.3 ± 7.3

Figure S1. IPSS change over the study period for a) IPSS total, b) IPSS obstructive, c) IPSS irritative, and d) IPSS QoL.

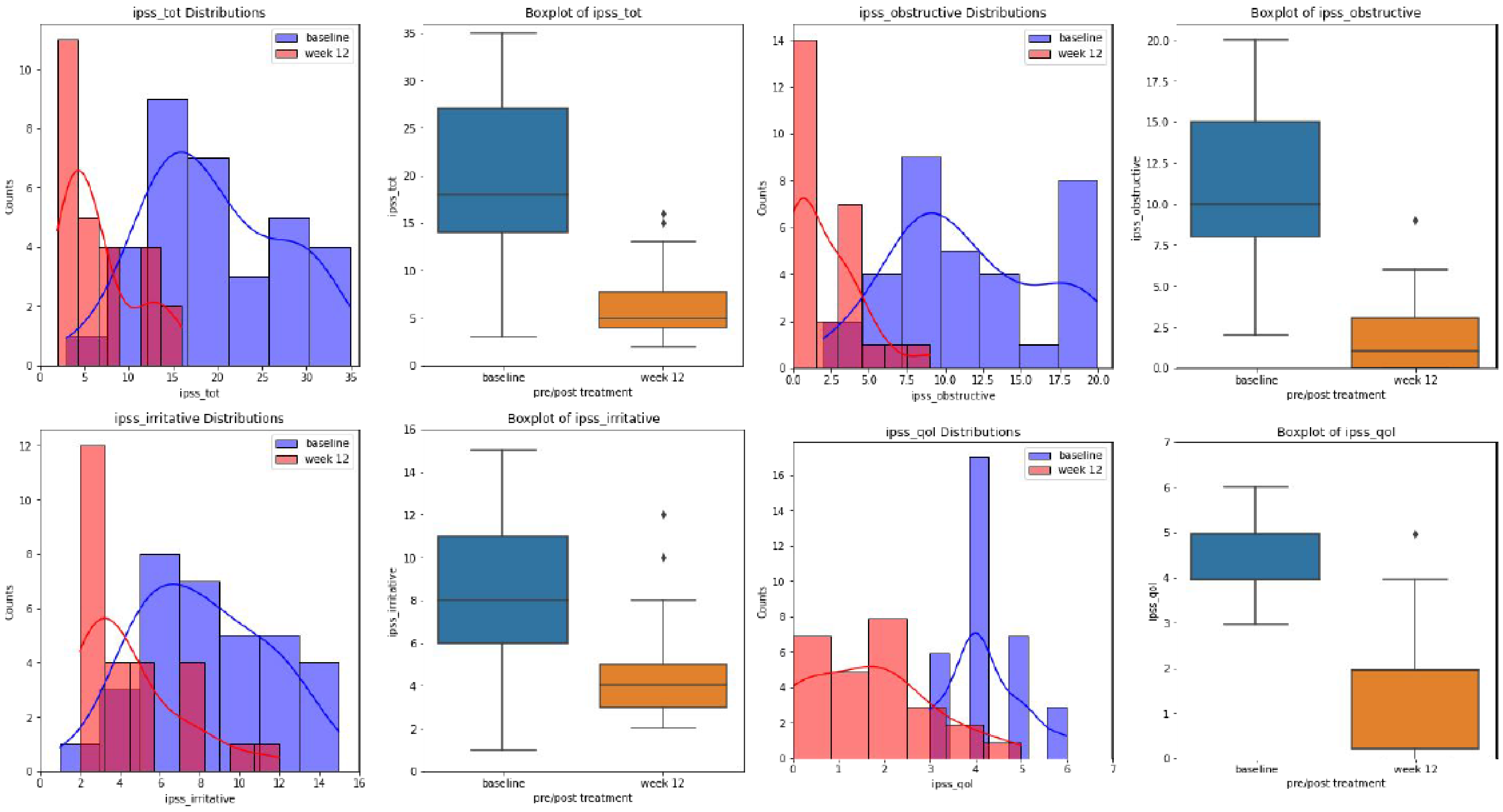

Figure S2. Changes in IPSS and Qmax correlation for a) IPSS total, b) IPSS obstructive, c) IPSS irritative, and d) IPSS QoL

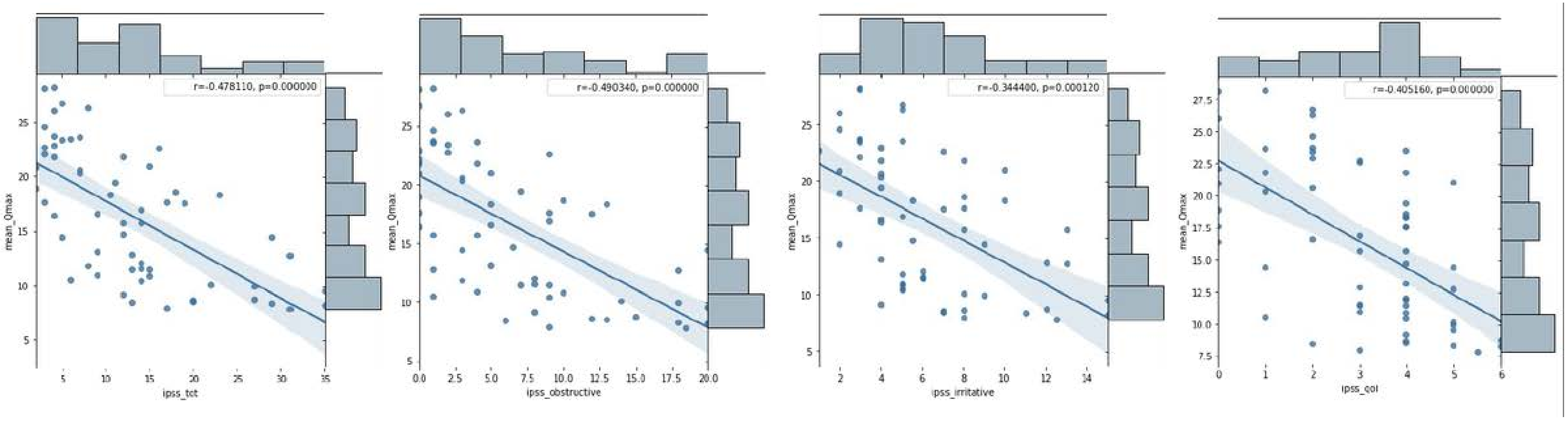

Figure S3. Qmax correlation stratified by severity of preoperative IPSS

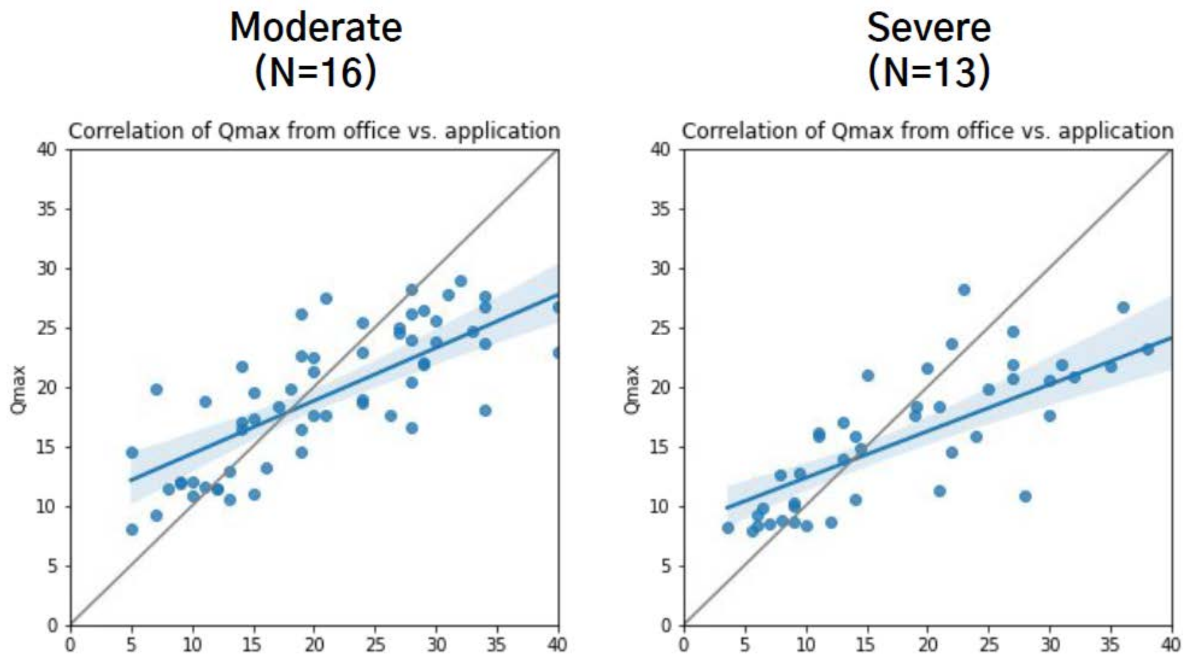

Supplement: Multimedia Appendix 2 [file jmir_v27i1e75313_app2.pdf]
